# The D614G mutation redirects SARS-CoV-2 spike to lysosomes and suppresses deleterious traits of the furin cleavage site insertion mutation

**DOI:** 10.1126/sciadv.ade5085

**Published:** 2022-12-23

**Authors:** Chenxu Guo, Shang-Jui Tsai, Yiwei Ai, Maggie Li, Eduardo Anaya, Andrew Pekosz, Andrea Cox, Stephen J. Gould

**Affiliations:** ^1^ Department of Biological Chemistry, Johns Hopkins University, School of Medicine, 725 North Wolfe Street, Baltimore, MD, 21205, USA.; ^2^ Department of Microbiology and Immunology, Johns Hopkins University, School of Public Health, 615 North Wolfe Street, Baltimore, MD 21205, USA.; ^3^ Department of Medicine, Department of Microbiology and Immunology, Johns Hopkins University, School of Medicine, 725 North Wolfe Street, Baltimore, MD, 21205, USA.

## Abstract

Severe acute respiratory syndrome coronavirus 2 (SARS-CoV-2) egress occurs by lysosomal exocytosis. We show that the Spike D614G mutation enhances Spike trafficking to lysosomes, drives Spike-mediated reprogramming of lysosomes, and reduces cell surface Spike expression by ~3-fold. D614G is not a human-specific adaptation. Rather, it is an adaptation to the earlier furin cleavage site insertion (FCSI) mutation that occurred at the genesis of SARS-CoV-2. While advantageous to the virus, furin cleavage of spike has deleterious effects on spike structure and function, inhibiting its trafficking to lysosomes and impairing its infectivity by the transmembrane serine protease 2(TMPRSS2)-independent, endolysosomal pathway. D614G restores spike trafficking to lysosomes and enhances the earliest events in SARS-CoV-2 infectivity, while spike mutations that restore SARS-CoV-2’s TMPRSS2-independent infectivity restore spike’s trafficking to lysosomes. Together, these and other results show that D614G is an intragenic suppressor of deleterious traits linked to the FCSI and lend additional support to the endolysosomal model of SARS-CoV-2 egress and entry.

## Teaser

D614G is an intragenic suppressor of deleterious traits caused by the FCSI mutation and furin cleavage of Spike.

## INTRODUCTION

COVID-19 (coronavirus infectious disease 2019) is caused by the enveloped virus severe acute respiratory syndrome coronavirus 2 (SARS-CoV-2) (*1*) (coronavirus.jhu.edu). Coronaviruses have the largest genomes of all RNA viruses, and SARS-CoV-2 encodes more than two dozen proteins, most of which are essential for virus replication (*2*, *3*). Of these, the spike protein is particularly critical because it mediates viral binding to SARS-CoV-2 receptors [angiotensin converting enzyme-2 (ACE2) (*1*, *4*–*7*), neuropilin-1 (*6*, *7*), and heparan sulfate (*8*, *9*)], catalyzes the fusion of viral and cellular membranes (*10*, *11*), is the sole antigen expressed by most SARS-CoV-2 vaccines (*12*), and is the primary target of antibody-based COVID-19 therapeutics (*13*, *14*).

Several lines of evidence indicate that coronavirus assembly occurs in the endoplasmic reticulum (ER)–Golgi intermediate compartment (ERGIC) (*15*–*17*). However, newly synthesized coronavirus particles do not appear to be released by the classic secretory pathway but are instead trafficked to lysosomes and/or lysosome-like compartments, where they accumulate until they are released en masse by an ADP-ribosylation factor-like 8b (Arl8b)-dependent pathway of lysosomal exocytosis (*18*, *19*). Expectedly, this lysosomal mode of viral egress coincides with extensive reprogramming of host lysosomes, as the virus converts them from degradative organelles to viral storage-and-release compartments. This process involves lysosome deacidification, lysosome exocytosis, lysosome clustering, lysosomal accumulation of KDEL receptors (*20*–*24*), aberrant secretion of ER-resident proteins and lysosomal hydrolases, impaired lysosomal uptake of endocytosed materials, and the accumulation of newly synthesized virus particles in large, distended lysosomes (*18*).

The lysosomal pathway of coronavirus egress is mirrored by the endolysosomal pathway of coronavirus entry (*25*–*38*). Hallmarks of this pathway include the appearance of coronavirus particles in lysosomes shortly after viruses are added to target cells, as well as requirements for lysosomal proteases, the vacuolar adenosine triphosphatase (V-ATPase), and lysosomal trafficking factors in the early stages of coronavirus infection. In the case of sarbecoviruses other than SARS-CoV-2, virus-receptor binding leads to virion endocytosis, followed by cathepsin-mediated cleavage of spike at a conserved arginine at the S1/S2 junction, a second cathepsin-mediated cleavage at the spike S′ site, and then spike-mediated fusion of viral and cell membranes. However, SARS-CoV-2 differs from the other sarbecoviruses in that ~50% of its spike proteins are cleaved at the S1/S2 junction before the release of SARS-CoV-2 virions, priming SARS-CoV-2 particles for rapid infection at the surface of cells that express TMPRSS2 or other cell surface serine proteases (*4*, *10*, *11*, *39*–*41*).

Cleavage of SARS-CoV-2 spike during its biogenesis is only possible because the near-immediate ancestor of SARS-CoV-2 acquired a 12-nucleotide-long/4–amino acid–long furin cleavage site insertion (FCSI) mutation (681PRRA684). This four-codon insertion created a furin protease cleavage site (RxxR|S) at the S1/S2 junction (681P**R**RA**R|S**686), allowing the Golgi-resident protease furin to cleave spike into S1 and S2 proteins. This cleavage potentiates spike-catalyzed fusion of virus and cell membranes independently of endolysosomal cathepsins, so long as the recipient cell expresses TMPRSS2 (or other cell surface serine protease).

Based on these considerations, the FCSI mutation can be seen as providing SARS-CoV-2 with the ability to infect cells by a new, TMPRSS2-dependent pathway of infectivity at the cell surface. This pathway is absolutely essential for respiratory transmission of SARS-CoV-2 (*1*, *6*, *7*, *10*, *11*, *42*–*49*) but the FCSI mutation also resulted in a large structural change to spike protein. This is evident in its cleavage into two separate polypeptides, S1 and S2. Although we do not know the structure of furin-cleaved spike, it is well-established that mutations almost always have deleterious traits (*50*–*53*). This appears to the be the case for the FCSI mutation, as it is already known to cause poor infectivity by the endolysosomal pathway of virus entry (*24*, *40*, *45*, *47*–*49*, *54*–*56*). This is evident in the poor replication of SARS-CoV-2 on Vero E6 and other cells that lack TMPRSS2 expression, as well as the rapid emergence of FCSI reversion mutants following serial passage of SARS-CoV-2 on these cell types. 

Shortly after its zoonotic leap to humans, SARS-CoV-2 acquired yet another strongly advantageous mutation in its spike gene, D614G (*57*). This mutation confers a pronounced increase in SARS-CoV-2 infectivity, as well as increased viral load and superior transmission (*57*–*63*). This increase in viral fitness appears to be at least partly driven by a D614G-mediated acceleration in the early phases of SARS-CoV-2 entry (*59*). Numerous studies have identified additional changes linked to the D614G mutation, including increased stability of the open or “up” conformation of the spike receptor-binding domain, altered ACE2 binding, enhanced spike processing, and improved postcleavage retention of S1 and greater thermal stability (*60*, *61*, *64*–*70*). Into this rich array of empirical observations, we show here that D614G redirects spike from the plasma membrane to lysosomes, restoring a robust lysosomal trafficking that was lost as a consequence of the FCSI mutation and furin cleavage of spike at the S1/S2 boundary. Furthermore, we show that spike protein mutations that restore robust infectivity by the TMPRSS2-independent infectivity do so by redirecting spike from the plasma membrane to lysosomes. These results shed new light on the early evolutionary genetics of SARS-CoV-2, highlight the need to better understand the effects of furin cleavage on spike structure, and indicate that a full understanding of spike biology requires identification of its lysosomal sorting receptor and its potential contributions to SARS-CoV-2 biogenesis, egress, and entry.

## RESULTS

### D614G drives a threefold reduction in cell surface spike expression

**Fig. 1.**
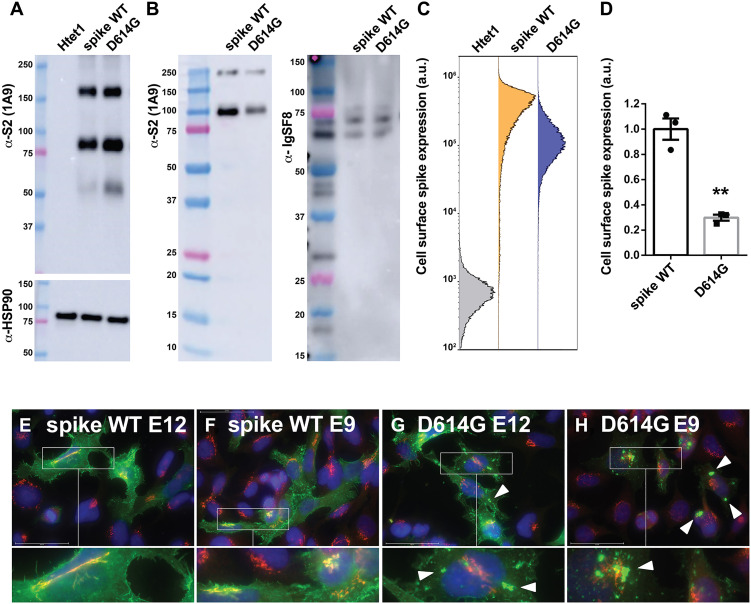
D614G decreases cell surface spike expression by ~3-fold. (**A**) Anti-spike immunoblot of cell lysates prepared from equal numbers of doxycycline-induced Htet1, Htet1/S^W1^, and HTet1/S^D614G^ cells, using a monoclonal antibody (1A9) that was originally raised against SARS spike but also binds a conserved epitope in the SARS-CoV-2 spike S2 domain, far from the site of the D614G mutation. These results are representative of three independent trials (*n* = 3). Molecular weight (MW) markers, from top, are 250, 150, 100, 75 (pink), 50, and 37 kDa. α-HSP90, heat shock protein 90 alpha family class A member 1. (**B**) Immunoblots of avidin affinity–purified proteins extracted from cell surface biotin-labeled HTet1/S^W1^ and HTet1/S^D614G^ cells, probed for (left) SARS-CoV-2 spike and (right) IgSF8. These results are representative of three independent trials (*n* = 3). MW markers, from top, are 250, 150, 100, 75 (pink), 50, 37, 25 (pink), 20, 15, and 10 kDa. (**C**) Flow cytometry histograms of doxycycline-induced HTet1 (gray), HTet1/S^W1^ (orange), and HTet1/S^D614G^ cells (blue) stained with Alexa Fluor 647 (A647) conjugates of the 1A9 anti-S2 antibody. These results are representative of three independent trials (*n* = 3). a.u., arbitrary units. (**D**) Bar graph of cell surface fluorescence from three independent trials, with error brackets denoting SEM, and ** referring to a Student’s *t* test *P* < 0.005 (*P* = 0.0013). (**E** to **H**) Immunofluorescence micrographs of doxycycline-induced (E and F) HTet1/S^W1^ cells and (G and H) HTet1/S^D614G^ cells stained human plasmas from patients with COVID-19 (E and G) E12 and (F and H) E9 to visualize the distribution of spike (green), the Golgi marker GM130 (red), and 4′,6-diamidino-2-phenylindole (DAPI) to mark the nucleus (blue). Scale bars, 50 μm (main images). White arrowheads denote the large, spike-containing structures of unknown identity. These results are representative of three independent trials (*n* = 3).

Viruses often evolve to reduce their cell surface expression, presumably because adaptive immune responses select for mutant viruses that are better able to escape immune surveillance (*71*–*73*). To determine whether the D614G mutation affects cell surface spike expression, we created HEK293-derived, tet-on cell lines that express WT and D614G forms of spike in response to doxycycline (Htet1/S^W1^ and Htet1/S^D614G^ cells, respectively), labeled their surface proteins by addition of sulfo–NHS (*N*-hydroxysuccinimide)–SS-biotin, then lysed the cells, purified all biotinylated surface proteins by avidin affinity chromatography, and interrogated them by immunoblot. These two cell lines expressed equivalent amounts of WT and D614G forms of spike (Fig. 1A), but the WT form of spike was far more abundant at the cell surface than the D614G form of spike, while both cell lines expressed equivalent cell surface levels of the endogenously expressed cell surface control protein immunoglobulin superfamily member 8 (IgSF8) (Fig. 1B). As an independent test of this same question, we performed cell surface anti-spike flow cytometry experiments, which revealed that the D614G mutation caused an ~3-fold reduction in the cell surface expression of spike [30% ± 2% (SEM), *P* = 0.0013] (Fig. 1, C and D).

Given that the WT and D614G forms of spike were expressed at similar levels, the ~3-fold reduction in cell surface spike expression caused by the D614G mutation is likely mediated by a substantial accumulation of spike in some intracellular organelle. To explore this possibility, we processed doxycycline-treated HTet1/S^W1^ and HTet1/S^D614G^ for immunofluorescence microscopy (IFM) using the human polyclonal anti-spike antibodies present in COVID-19 patient plasmas, together with antibodies specific for the Golgi marker GM130 (SARS spike is known to accumulate in the Golgi) (*74*). These experiments revealed that WT spike showed strong cell surface localization and substantial colocalization with GM130 (Fig. 1, E and F), whereas D614G spike displayed little, if any, colocalization with GM130, less plasma membrane staining, and significant accumulation in large, non-Golgi compartments of unknown identity (Fig. 1, G and H).

### D614G redirects spike from the plasma membrane to lysosomes

**Fig. 2.**
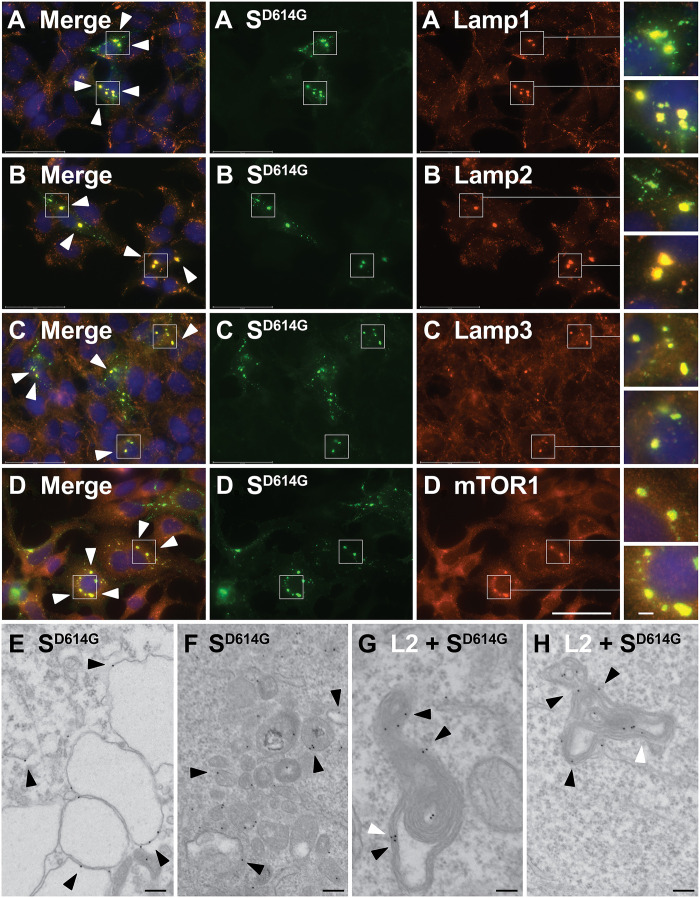
Human cells traffic spike to lysosomes. (**A** to **D**) Immunofluorescence micrographs of doxycycline-induced HTet1/S^D614G^ cells that had been fixed, permeabilized, and stained using anti-spike immune sera (green), DAPI (blue), and antibodies (red) specific for (A) Lamp1, (B) Lamp2, (C) Lamp3, or (D) mTOR1. White arrowheads denote the positions of spike-containing, lysosome-related structures. Scale bar, 50 μm (main images). Outsets show detail in areas of particular interest. Scale bar, 4 μm (outset). These results are representative of three independent trials (*n* = 3). (**E** to **H**) Immunogold electron micrographs of doxycycline-induced HTet1/S^D614G^ cells processed for electron microscopy using (E and F) affinity-purified anti-spike antibodies specific for its C-terminal 14 amino acids (12-nm gold) or (G and H) both anti-spike C14 antibodies (12-nm gold) and anti–Lamp2 (L2) antibodies (6-nm gold). Black arrowheads denote the positions of 12-nm gold directed against spike, and white arrowheads denote the positions of 6-nm gold directed against Lamp2. Scale bars, 200 nm. These immunogold labeling results are representative of two independent trials.

To identify these spike-containing compartments, we co-stained doxycycline-induced HTet1/S^D614G^ cells with antibodies specific to known organelle marker proteins and the human polyclonal anti-spike antibodies present in COVID-19 patient plasmas. S^D614G^ displayed no colocalization with markers of the ER [calnexin and heat shock protein family A (Hsp70) member 5 (BiP)], ERGIC (ERGIC53 and ERGIC3), Golgi (GM130), endosome [early endosome antigen 1 (EEA1)], or plasma membrane (CD81) (fig. S1). In contrast, spike displayed substantial colocalization with all lysosome membrane proteins tested, including LAMP1, LAMP2, LAMP3, and mammalian target of rapamycin (mTOR) (Fig. 2, A to D).

These IFM results establish that spike is a lysosomal protein but did not differentiate between spike engulfment within lysosomes or spike accumulation in the limiting membrane of lysosomes. To address this issue, we performed immunogold electron microscopy on doxycycline-induced HTet1/S^D614G^ cells using an affinity-purified anti-spike antibody directed against its C-terminal 14 amino acids (fig. S2). Spike labeling (12-nm gold) was detected only at the limiting membrane of lysosome-like structures, including the membranes of large empty lysosomes (Fig. 2E), small, electron-dense lysosomes (Fig. 2F), and multilamellar endo/lysosomal compartments (Fig. 2, G and H), demonstrating that spike is not being sent to the lumen of lysosomes for subsequent degradation. Consistent with this interpretation, IFM of doxycycline-induced HTet1/S^D614G^ cells incubated with the PIKfyve inhibitor vacuolin-1 (which causes lysosomes to swell dramatically) confirmed that spike was embedded in the limiting membrane of lysosomes, along with Lamp2 (fig. S3).

**Fig. 3.**
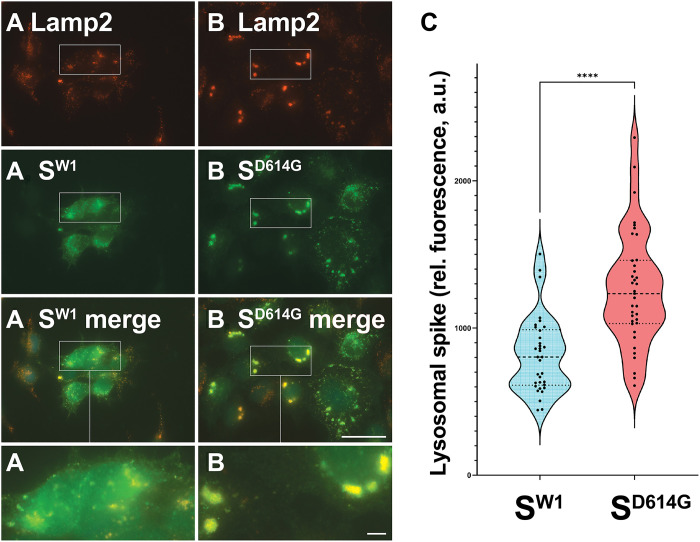
D614G increases spike trafficking to lysosomes. (**A** and **B**) Fluorescence micrographs of doxycycline-induced (A) HTet1/S^W1^ and (B) HTet1/S^D614G^ cells that had been fixed, permeabilized, and stained using (green) affinity-purified anti-spike antibodies specific for the C-terminal 14 amino acids of spike, (blue) DAPI, and (red) anti-Lamp2 antibodies. Scale bar, 50 μm. Outsets (3.2-fold higher magnification) show detail in areas of particular interest. Scale bar, 4 μm (outset). These results are representative of three independent trials (*n* = 3). (**C**) Violin plot showing the amount of lysosome-localized spike fluorescence, quantified from >30 immunofluorescence micrographs of doxycycline-induced HTet1/S^W1^ cells and HTet1/S^D614G^ cells, following staining with the S.C14 affinity-purified antibody and a monoclonal antibody to Lamp2. Dashed line denotes the median, dotted lines defining the middle 50% of samples, and **** denotes Student’s *t* test *P* = 2.4 × 10^−7^. Similar results were observed in >10 trials.

Having established that the large organelles containing spike were lysosomes, we next set out to quantify the effect of the D614G mutation on the lysosomal accumulation of spike. Toward this end, we stained doxycycline-induced HTet1/S^W1^ and HTet1/S^D614G^ cells with the antibody that binds the cytoplasmically oriented, C-terminal 14 amino acids of spike (C14 antibody; fig. S2), as well as with a monoclonal antibody to Lamp2, then collected numerous IFM images (Fig. 3, A and B), and quantified the amount of WT and D614G spike that colocalized with Lamp2. Using data from ≥30 independent images collected from each cell line, we observed that the D614G mutation caused a ~1.5-fold increase in the lysosomal localization of spike (1.5×; *P* = 2 × 10^−7^), represented here by violin plot (Fig. 3C).

### D614G redirects spike to lysosomes during SARS-CoV-2 infection

**Fig. 4.**
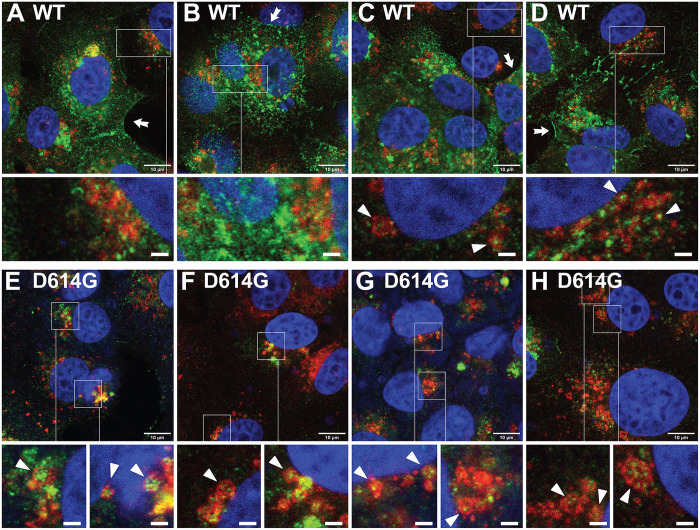
D614G alters spike trafficking in virus-infected cells. Confocal fluorescence micrographs of Vero/TMPRSS2 cells infected with (**A** to **D**) HP76, a WT strain of SARS-CoV-2 that expresses the D614 form of spike, and (**E** to **H**) HP7, a D614G strain of SARS-CoV-2. Cells were infected with equal amounts of virus (MOI of 10), incubated for 18 hours, then fixed, permeabilized, and stained with affinity-purified antibodies specific for the C-terminal 14 amino acids of spike (green), a monoclonal anti-Lamp2 antibody (red), and DAPI (blue). White arrows point to the plasma membrane accumulation of spike in HP76-infected cells. White arrowheads point to spike-positive virus particles within Lamp2-positive lysosomes. Insets (3.2-fold higher magnification) show greater detail in areas of particular interest. Scale bars, 10 μm (main images) and 2 μm (magnified outsets). These results are representative of four independent trials (*n* = 4).

We next tested whether the D614G affected the intracellular sorting of spike in the context of a virus infection. Specifically, we infected Vero E6/TMPRSS2 cells with either D614 (HP76) or G614 (HP7) strains of SARS-CoV-2 [multiplicity of infection (MOI) of 10], incubated the cells for 18 hours, and then processed the cells for confocal IFM using the antibody that binds the C-terminal 14 amino acids of spike (C14 antibody) and a monoclonal antibody specific for Lamp2. Cells infected with the WT spike strain of SARS-CoV-2 showed substantial spike staining at the plasma membrane and various other compartments but relatively little colocalization of spike with the lysosome marker Lamp2 (Fig. 4, A to D). Furthermore, only small numbers of spike vesicles/virions were detected within the lysosomes of these cells (arrowheads in higher magnification outsets). In contrast, cells infected with the G614 strain of SARS-CoV-2 (*75*) localized spike almost exclusively to perinuclear structures, near or coincident with Lamp2 staining (Fig. 4, E and F). Moreover, the lysosomes of HP7-infected cells were often enlarged and packed with spike-positive virus particles in their lumen (see higher magnification outsets), consistent with the idea that the lysosomal delivery of spike contributes to the biogenesis of virus particles, as shown previously by Ghosh *et al.* (*18*). It should also be noted that infection with HP7 induced the clustering of lysosomes in the perinuclear region of infected cells, similar to what we observed in cells expressing high levels of S^D614G^ (Fig. 2).

**Fig. 5.**
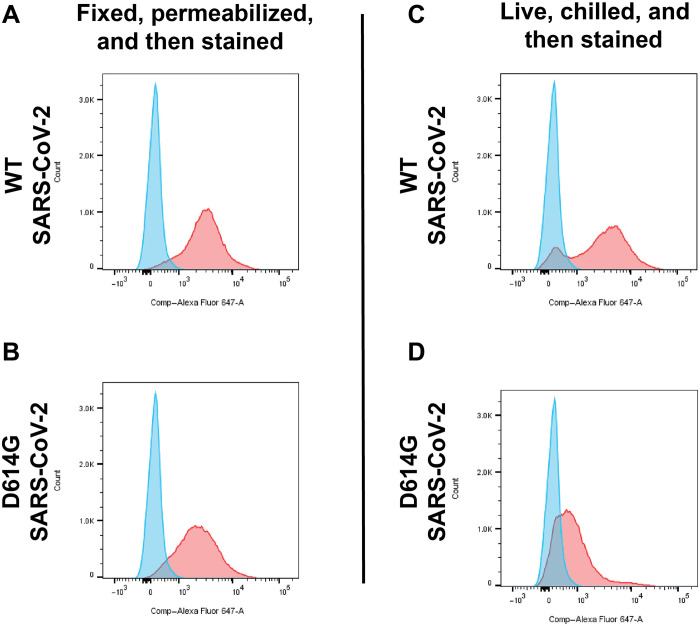
The D614G reduces the cell surface spike expression in virus-infected cells. Anti-spike flow cytometry data collected from Vero E6/TMPRSS2 cells, either (blue) mock infected or (red) infected 18 hours earlier at an MOI of 1. Cells were infected with (**A** and **C**) a strain of SARS-CoV-2 that expresses the WT form of spike or (**B** and **D**) a strain of SARS-CoV-2 that expresses the D614G form of spike and then, 18 hours later, were examined by flow cytometry of (**A** and **B**) cells that had been fixed and permeabilized, to detect the overall level of spike expression, or (**C** and **D**) cells that had been chilled to 4°C, to detect the levels of spike expressed at the cell surface. These experiments were performed once.

To determine whether the enhanced lysosomal accumulation of spike correlated with reduced spike expression at the cell surface, we again infected Vero E6/TMPRSS2 cells with WT (HP76) and D614G (HP7) strains of SARS-CoV-2 (MOI of 1), although, in this case, the cells were interrogated by flow cytometry. Using an Alexa Fluor 647 (A647) conjugate of anti-S2 1A9 monoclonal antibody described previously (Fig. 1), we observed that anti-spike flow cytometry of fixed and permeabilized cells confirmed that the amount of spike expressed in these cells was roughly similar (Fig. 5, A and B). However, when we performed flow cytometry on live cells that had been chilled and labeled to detect only cell surface–exposed spike proteins (though subsequently fixed), we found that the D614G mutation resulted in a ~3-fold decrease in the display of spike at the cell surface (Fig. 5, C and D). Thus, the D614G mutation had similar effects on the subcellular trafficking of spike in SARS-CoV-2–infected cells and in 293 cells expressing spike alone without any other viral proteins.

### Spike trafficking to lysosomes is mediated by its extracellular domain

**Fig. 6.**
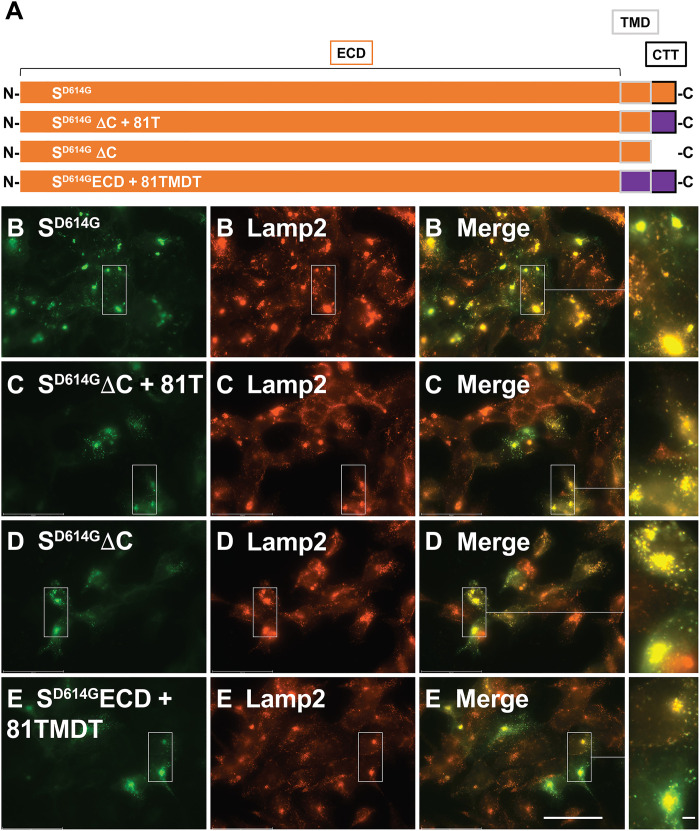
Spike trafficking to lysosomes is mediated by its ECD. (**A**) Line diagram of spike proteins showing the position of its ECD (bracket), transmembrane domain (TMD; gray outline), and C-terminal tail (CTT; black outline), with orange denoting spike sequences and purple denoting CD81 sequences. (**B** to **E**) Fluorescence micrographs of doxycycline-induced (B) HTet1/S^D614G^ cells, (C) HTet1/S^D614G^ΔC + 81T cells, (D) HTet1/S^D614G^ΔC cells, and HTet1/S^D614G^ΔC + 81TMDT cells that had been processed for IFM using with anti-spike immune sera (green), DAPI (blue) and anti-Lamp2 antibody (red). Scale bars, 50 μm (main image) and 4 μm (magnified outset). These results are representative of two independent trials (*n* = 2).

The preceding observations demonstrate that spike is a type-1 lysosome membrane protein and that the D614G mutation enhances spike accumulation in lysosomes. Given that most other type-1 lysosome membrane proteins carry short lysosome targeting signals matching the dileucine (DxxLL or [DE]xxxL[LI]) or tyrosine-based (YxxØ) peptides that interact with clathrin adaptor protein complexes (*76*), we searched the spike C-terminal tail for matches to these lysosomal sorting motifs. However, the C-terminal tail of spike (-KFDEDDSEPVLKGVKLHYT_COOH_) lacks similarity to known lysosomal targeting signals. Even so, the presence of lysosomal sorting signals in the C-terminal tail of type-1 proteins is so ubiquitous that we tested whether loss of its tail blocked spike trafficking to lysosomes. Specifically, we followed the expression and lysosomal trafficking of S^D614G^ proteins (S^D614G^ΔC+81T and S^D614G^ΔC) that either replaced spike’s tail with that of CD81, a plasma membrane protein (-KRNSSVY_COOH_), or replaced it with a single lysine residue and found that both were still localized to lysosomes (Fig. 6, A to D). Furthermore, when we expressed the spike extracellular domain (ECD; amino acids 1 to 1187) fused to the N terminus of an inert, type-1 plasma membrane anchor [the transmembrane domain and tail sequence of human CD81 (-SGKLYLIGIAAIVVAVIMIFEMILSMVLCCGIRNSSVY_COOH_)], we found that the resulting protein (S^D614G^ECD + 81TMDT) also colocalized with Lamp2 (Fig. 6, A and E). These data show that the lysosomal sorting information of spike resides in the extracellular region of the protein. Consistent with these observations, we observed that spike trafficking to lysosomes was unaffected by inhibitors of clathrin-dependent, clathrin-independent, and microtubule-dependent protein traffic, namely the dynamin inhibitor dynasore, the clathrin inhibitor pitstop2, and the microtubule-depolymerizing agent nocodazole (fig. S4) (*77*–*81*).

### D614G is not a human-specific adaptation

It is well established that the D614G mutation arose under strong positive selection (*57*). This indicates that D614G mutation was selected as an adaptation to a relatively recent change in either the environment or the genetics of SARS-CoV-2. The most obvious change in the environment of SARS-CoV-2 is its new human host so we testedwhether the D614G-mediated shift in spike protein trafficking was a human-specific adaptation. Specifically, we measured the total expression and the cell surface expression of WT and D614G spike proteins in cells from a distantly related mammal. Humans lie within the clade of Euarchontoglires (primates, rodents, and lagomorphs), which is within the much larger clade of Eutherians, whereas the Metatheria (opossum, kangaroo, and other marsupials) define a separate, evolutionarily distant clade of mammals. Thus, if D614G has the same effect on spike trafficking in a marsupial cell line, it would dispel the notion that D614G is a human-specific adaptation. We therefore created derivatives of the opossum cell line OK (*82*) that express WT and D614G forms of spike in response to doxycycline (OK/S^W1^ and OK/S^D614G^ cells), cultured them in the presence of doxycycline, and assayed them for their total expression and cell surface expression of spike (fig. S5). The D614G mutation caused the same ~3-fold reduction in cell surface expression of spike in marsupial cells as it had human/Eutherian cells, indicating that the D614G-mediated shift in spike protein trafficking was (i) independent of the cell type and mammalian species in which it was expressed, (ii) intrinsic to the spike protein itself, and (iii) not an adaptation to SARS-CoV-2’s new environment of the human cell.

### D614G suppresses an FCSI-linked, furin-mediated defect in spike protein trafficking

With no evidence that D614G was an evolutionary adaptation to its new human environment, we turned to the only remaining parsimonious explanation, which is that D614G arose as an evolutionary adaptation to its genetics and, more specifically, to a major recent change in the spike gene/protein itself. As for what this pre-D614G change might be, previous studies have established that the near-immediate ancestor of SARS-CoV-2 acquired a 12-nucleotide/four–amino acid FCSI at the S1/S2 junction of the spike gene/protein (*4*, *11*, *39*–*41*). Furthermore, there are a few reasons to presume that the FCSI mutation, while essential to SARS-CoV-2’s ability to efficiently infect TMPRSS2-expressing cells and spread by respiratory transmission, was also linked to deleterious traits. This proposition is supported by a number of considerations, not least of which is that mutations are almost always deleterious (*50*, *52*, *53*). The specifics of FCSI mutation make it almost inconceivable that it would not engender large changes in spike structure and function, as it inserted four amino acids that results in furin-mediated cleavage of spike into two polypeptides. Furthermore, there is already strong empirical data showing that the FCSI is linked to deleterious traits, as SARS-CoV-2 displays only poor infectivity of Vero E6 cells, and that passage of SARS-CoV-2 on Vero E6 cells leads to the rapid rise of mutant viruses that rapidly outcompete WT SARS-CoV-2 (*24*, *40*, *45*, *47*–*49*, *54*–*56*), such as the ΔN679-A688 and ΔR683-S689 mutations that delete the FCSI (*24*).

**Fig. 7.**
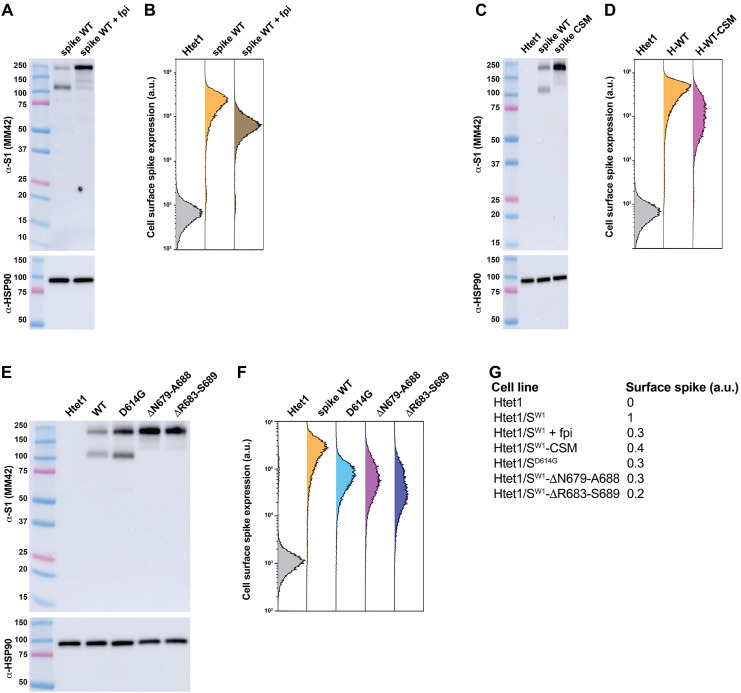
Furin and the FCSI insertion caused a threefold increase in cell surface spike expression. (**A**) Anti-spike and anti-HSP90 immunoblots of cell lysates prepared from doxycycline-induced HTet1/S^W1^ cells that had been incubated with either control medium or with medium containing the furin protease inhibitor (fpi) decanoyl-RVKR-CMK (+fpi). MW markers, from top, are 250, 150, 100, 75 (pink), 50, 37, 25 (pink), 20, 15, and 10 kDa. (**B**) Histograms of cell surface anti-spike fluorescence measurements captured by flow cytometry of doxycycline-induced HTet1 cells (gray), HTet1/S^W1^ (gold), and HTet1/S^W1^ cells (brown) that had been incubated with the furin protease inhibitor (+fpi) decanoyl-RVKR-CMK. (**C**) Anti-spike and anti-HSP90 immunoblots of cell lysates prepared from equal amounts of doxycycline-induced HTet1, HTet1/S^W1^, and HTet1/S^W1^-CSM cells. MW markers, from top, are 250, 150, 100, 75 (pink), 50, 37, 25 (pink), 20, and 15 kDa. (**D**) Histograms of cell surface anti-spike fluorescence intensity data obtained by flow cytometry of doxycycline-induced HTet1 (gray), HTet1/S^W1^ (gold), and HTet1/S^W1^-CSM cells (purple). (**E**) Anti-spike and anti-HSP90 immunoblots of cell lysates prepared from doxycycline-induced HTet1, HTet1/S^W1^ (WT), HTet1/S^D614G^ (D614G), HTet1/S^W1^-ΔN679-A688 cells (ΔN679-A688), and HTet1/S^W1^-ΔR683-S689 cells (ΔR683-S689). MW markers, from top, are 250, 150, 100, 75 (pink), 50, 37, 25 (pink), 20, and 15 kDa. (**F**) Histograms of cell surface anti-spike fluorescence measurements captured by flow cytometry of doxycycline-induced HTet1 cells (gray), HTet1/S^W1^ cells (orange), HTet1/S^D614G^ cells (blue), HTet1/S^W1^-ΔN679-A688 cells (dark purple), and HTet1/S^W1^-ΔR683-S689 (lavender). (**G**) Relative cell surface expression of spike in each of the cell lines tested in these experiments. Similar results were observed in three independent trials (*n* = 3).

In light of these considerations, we tested whether furin activity and the FCSI mutation disrupt the trafficking of WT spike to lysosomes. HTetZ/S^W1^ cells were incubated in the presence or absence of the cell-permeable furin protease inhibitor decanoyl-RVKR-CMK (50 μM), then induced to express spike by addition of doxycycline, and, a day later, interrogated by anti-spike immunoblot of whole-cell lysates and by anti-spike cell surface flow cytometry to test whether furin activity led to a threefold increase in the cell surface expression of spike. It did (Fig. 7, A, B, and G), providing strong evidence that the FCSI mutation leads to a furin-mediated disruption of spike trafficking to lysosomes. If this interpretation is correct, then we should see a compensatory ~3-fold drop in cell surface expression for S^W1^-cleavage site mutation (CSM), in which uncharged amino acids were substituted for the three arginine residues within the furin cleavage site (682RRAR685-to-682GSAG68). We did see this drop (Fig. 7, C, D, and G), adding to the evidence that the FCSI mutation and furin cleavage caused a defect in spike structure that impairs its intracellular trafficking.

**Fig. 8.**
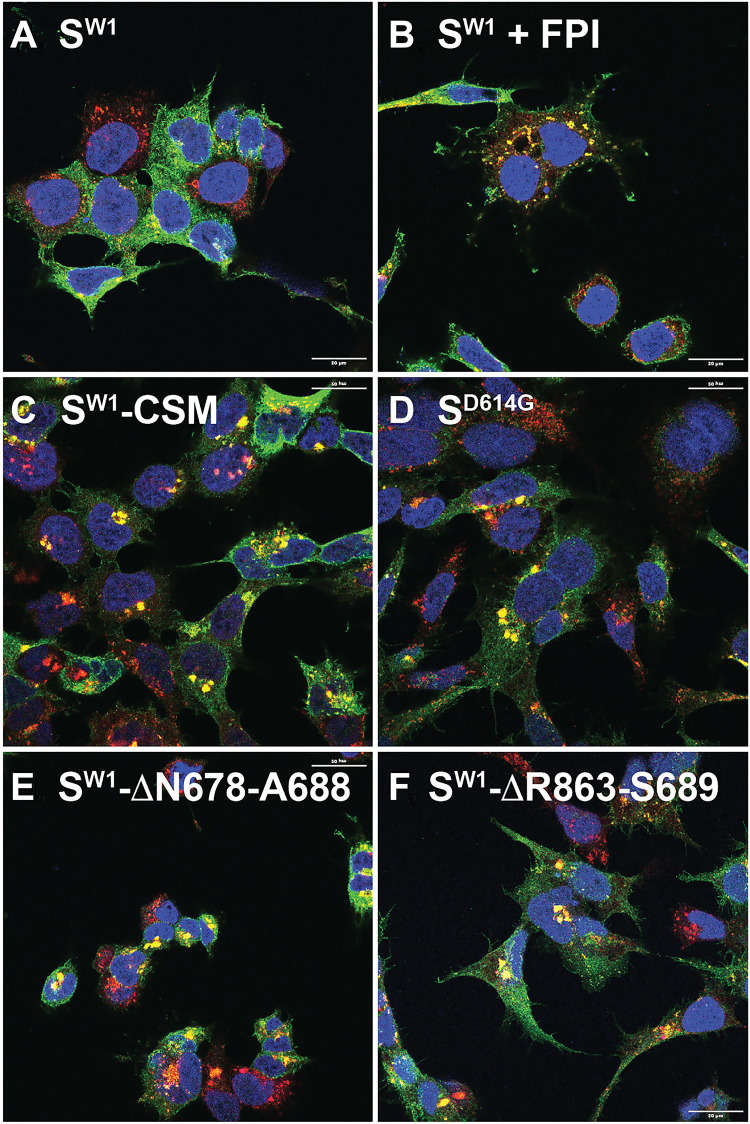
Furin and the FCSI mutation disrupt spike trafficking to lysosomes. Confocal immunofluorescence micrographs of doxycycline-induced cells that had been fixed, permeabilized, and stained using anti-spike antibodies specific for the C-terminal 14 amino acids of spike (green), DAPI (blue), and antibodies specific for Lamp2 (red). The cells shown here are doxycycline-induced (**A**) HTet1/S^W1^ (**B**) HTet1/S^W1^ cells incubated with the furin protease inhibitor (FPI), (**C**) HTet1/S^W1^-CSM, (**D**) HTet1/S^D614G^, (**E**) HTet1/S^W1^-ΔN679-A688, and (**F**) HTet1/S^W1^-ΔR683-S689. Scale bars, 50 μm. These results are representative of two independent trials.

In light of these results, the fact that the D614G mutation causes a ~3-fold reduction in cell surface spike (Figs. 1 and 7, E, F, and G) provides solid evidence that the D614G mutation functions as an intragenic suppressor of the earlier FCSI mutation. To further test this hypothesis, we asked whether the FCSI reversion mutants that are selected by passage on TMPRSS2-deficient cells share the same phenotype as the FCSI suppressor mutation and restore spike trafficking to lysosomes. We observed that they do , as both the ΔN679-A688 and ΔR683-S689 reversion mutants decrease the cell surface expression of spike (Fig. 7, E, F, and G). Consistent with these results and interpretations, interrogation of these cells by confocal IFM indicated that these changes are reflected in complimentary changes in spike trafficking to lysosomes. Specifically, we observed that WT spike was distributed throughout the cell surface and in many intracellular compartments, only few of which colocalized with Lamp2 (Fig. 8A), whereas WT Spike displayed strong colocalization with Lamp2 in cells treated with the furin protease inhibitor decanoyl-RVKR-CMK (50 μM) (Fig. 8B), when the arginines of the furin site were eliminated (Fig. 8C), when the D614G mutation was present (Fig. 8D), or when spike carried the ΔN679-A688 and ΔR683-S689 reversion mutations (Fig. 8, E and F, respectively).

### Spike mediates coronavirus reprograming of lysosomes

The preceding results demonstrate that the FCSI mutation disrupted spike protein trafficking to lysosomes and that D614G acts as an intragenic suppressor of this phenotype. To test whether the D614G mutation might enhance other spike functions, we tested (i) whether spike expression mediates any of the lysosomal abnormalities that are hallmarks of betacoronavirus infection and (ii) whether the D614G mutation enhances any of these phenotypes. As noted previously, betacoronaviruses such as SARS-CoV-2 convert lysosomes from degradative compartments to viral storage and egress compartments, and are released by an arl8b-dependent pathway of lysosomal exocytosis (*18*, *19*). These studies also established that the lysosomal hallmarks of virus infection are the work of specific viral proteins, as orf3a expression on its own induces (i) lysosome deacidification, (ii) lysosome exocytosis, and (iii) aberrant secretion of lysosome hydrolases. We therefore tested whether expression of spike might be responsible for one or more of the other virus-induced lysosomal phenotypes, which include (iv) lysosome clustering, (v) redistribution of KDEL receptors from the Golgi to lysosomes, (vi) a concomitant release of resident ER enzymes that carry the KDEL ER retrieval signal, and (vii) a defect in transfer of endocytosed materials from endosomes to lysosomes (*18*, *19*).

**Fig. 9.**
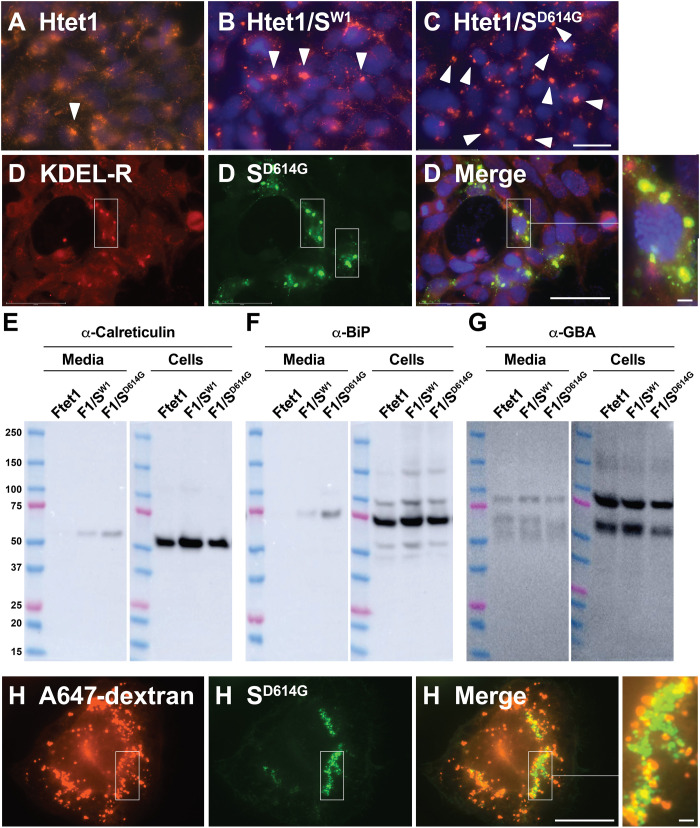
Spike disrupts lysosome and ER homeostasis. (**A** to **C**) Fluorescence micrographs of doxycycline-induced HTet1, HTet1/S^W1^, and HTet1/S^D614G^ cells labeled with DAPI (blue) and a monoclonal antibody specific for Lamp2 (red). White arrowheads denote highlight the positions of large clusters of lysosomes. Scale bar, 50 μm. (**D**) Fluorescence micrographs of doxycycline-induced HTet1/S^D614G^ cells that had been fixed, permeabilized, and stained using anti-spike immune sera (green), DAPI (blue), and anti-KDEL antibodies (red). Scale bar, 50 μm. Outset (3.2-fold higher magnification) shows detail in areas of particular interest. Scale bar, 4 μm. (**E** to **G**) Immunoblot of (left panels) secreted proteins and (right panels) cell lysates, interrogated using antibodies specific for (D) calreticulin, (E) BiP/GRP78, and (F) β-glucocerebrosidase (GBA). Quantitation of immunoblots from two independent trials revealed that the D614G mutation resulted in a ~4-fold increase in the secretion of calreticulin and BiP relative to WT spike. MW size markers, from top, are 250, 150, 100, 75 (pink), 50, 37, 25 (pink), 20, and 15 kDa. (**H**) Fluorescence micrographs of HTet1/S^D614G^ cells that had been incubated with A647-dextran, then washed, incubated for an additional 3 hours in normal medium, then fixed, permeabilized, and stained using (green) anti-spike immune sera and (red) antibodies to detect (G) Lamp2 or (H) the KDEL receptor. Scale bar, 50 μm (main image). These results are representative of two independent trials.

Lysosome clustering was measured by incubating HTet1, HTet1/S^W1^, and HTet1/S^D614G^ cells in doxycycline-containing medium overnight, then fixing the cells, and processing them for IFM using antibodies specific for Lamp2 to visualize the lysosomes and with 4′,6-diamidino-2-phenylindole (DAPI) to visualize the nucleus. We collected multiple images of each cell line and then processed these images to determine the number of large lysosome clusters (>2 μm in diameter) per number of nuclei across a minimum of 10 images. This allowed us to calculate a lysosome clustering index (LCI) for each cell line (LCI = [number of clustered lysosomes]/[number of nuclei]). As expected, lysosome clustering was infrequent in doxycycline-treated HTet1 cells (Fig. 9A), which exhibited an LCI of only 0.04 (40 of 970). Expression of WT spike clearly induced the clustering of lysosomes, as doxycycline-treated HTet1/S^W1^ cells (Fig. 9B) had an LCI of 0.14 (159 of 1187), much higher than the LCI of our control cell line. Cells expressing the D614G form of spike (Fig. 9C) displayed a further ~3-fold increase in lysosomal clustering, as their LCI was 0.43 (507 of 1192). In conclusion, WT spike was sufficient to induce lysosome clustering and D614G spike did so ~3-fold more efficiently.

We next tested whether spike expression is sufficient to induce the lysosomal accumulation of KDEL receptors. KDEL receptors are a trio of G protein–coupled receptors that perform a wide array of functions, including the retrieval of KDEL-containing ER-resident proteins back to the ER via COPI-mediated retrograde traffic (*20*–*23*). To assess their distribution in spike-expressing cells, doxycycline-induced HTet1/S^D614G^ cells were processed for IFM using antibodies specific for spike and KDEL receptors, which revealed that spike expression led to the lysosomal accumulation of KDEL receptors (Fig. 9D). To determine whether this leads to the aberrant secretion of KDEL-containing ER-resident enzymes seen in virus-infected cells (*18*), we generated Tet-on, spike-expressing derivatives of 293F cells (FTet1, FTet1/S^W1^, and FTet1/S^D614G^ cells), which can be grown in chemically defined medium (CDM) free of exogenous protein, greatly simplifying the analysis of protein secretion in response to spike. These FTet1, FTet1/S^W1^, and FTet1/S^D614G^ cells were cultured in CDM supplemented with doxycycline, followed by collection of cells and conditioned medium, and interrogated by immunoblot using antibodies specific for KDEL-containing resident ER proteins calreticulin and BiP and the lysosomal enzyme β-glucocerebrosidase (GBA) (Fig. 9, E to G). Neither of the KDEL proteins was secreted by FTet1 cells, while both were secreted in response to WT spike. We found that the D614G form of spike drove ~3-fold higher levels of calreticulin and BiP secretion, consistent with the notion that D614G mutation restores a wide range of spike-mediated processes. As for the specificity of this effect, we found that spike expression had no effect on the secretion of a soluble lysosomal enzyme. Thus, it seems clear that orf3a-induced lysosomal exocytosis and spike-induced secretion of ER-resident enzymes are separable phenotypes mediated by the specific actions of these two viral proteins (*18*, *19*).

We also used the tools available to us to test whether spike-containing lysosomes might be unable to take up endocytosed materials from endosomes, as was observed in virus-infected cells. Toward this end, we incubated HTet1/S^D614G^ cells in medium containing a fluorescently labeled dextran (A647-dextran), then washed the cells, and incubated them for an additional 3 hours, which is more than enough time to chase the endocytosed A647-dextrans to lysosomes. The cells were then fixed and processed for IFM, which revealed that spike-expressing cells were able to endocytose fluorescent dextrans but unable to transfer them from endosomes to spike-containing lysosomes (Fig. 9H).

## DISCUSSION

This report establishes that mammalian cells traffic SARS-CoV-2 spike to lysosomes and the plasma membrane, the D614G mutation redirects spike from the plasma membrane to lysosomes, the earlier FCSI mutation disrupts spike trafficking to lysosomes, and the D614G acts as an intragenic suppressor of deleterious traits conferred by the FCSI mutation. Furthermore, our data provide the first clear evidence that spike expression, on its own, contributes to the reprogramming of lysosome structure and function that are hallmarks of betacoronavirus-infected cells, demonstrating that spike does more than just bind receptors and catalyze membrane fusion. However, the most topical aspect of our study is the story it tells about the early evolutionary-genetic history of SARS-CoV-2, the impact of the FCSI mutation on spike structure and function, and the rise of D614G as an adaptation to its deleterious side effects.

### D614G is an intragenic suppressor of the FCSI mutation I: Effect on spike protein trafficking

Several lines of evidence indicate that D614G is an intragenic suppressor of an FCSI-linked defect in spike protein trafficking and, by deduction, spike protein structure. These observations include the following:

1) Furin-mediated cleavage of spike causes a pronounced defect in spike trafficking to lysosomes that leads to a ~3-fold increase in the cell surface expression of spike.

2) The D614G mutation restored spike trafficking to lysosomes, leading to a ~3-fold drop in its expression at the cell surface.

3) These D614G-mediated effects on spike protein trafficking were phenocopied by elimination of furin site arginines (CSM; 682RRAR685-to-682GSAG68).

4) These D614G-mediated effects on spike protein trafficking were also phenocopied by FCSI reversion mutants that arose spontaneously by growing SARS-CoV-2 on Vero E6 cells (ΔN679-A688 and ΔR683-S689) (*24*).

5) The D614G mutation had the same effect on spike protein trafficking in the marsupial cell line OK, refuting the hypothesis that D614G’s effect on spike protein trafficking was a human-specific adaptation.

### D614G is an intragenic suppressor of the FCSI mutation II: Effect on early/endolysosomal infectivity

In addition to disrupting the lysosomal sorting of spike, the FCSI mutation causes a defect in early stages of infectivity that is also suppressed by the D614G mutation. This can be seen in the following observations:

1) SARS-CoV-2 infects the TMPRSS2-deficient VeroE6 cell line only poorly (*10*, *24*, *40*, *45*–*49*, *54*–*56*, *83*).

2) This defect is caused by furin-mediated cleavage of spike and is manifested in the earliest phases of infection (*47*, *48*).

3) This early, furin-mediated defect in infectivity is suppressed by the D614G mutation (*59*).

4) Passage of SARS-CoV-2 on Vero E6 cells leads to the rapid emergence of SARS-CoV-2 viruses that replicate much more efficiently (*24*, *45*).

5) These fast-growing mutant strains of SARS-CoV-2 include some that carry FCSI reversion mutations (e.g., ΔN679-A688 and ΔR683-S689) (*24*, *45*).

6) These same mutations redirect spike from the plasma membrane to lysosomes, reducing cell surface spike expression by ~3-fold.

### Implications for SARS-CoV-2 evolution

The simplest interpretation of these observations is that the early prehistory and history of SARS-CoV-2 genetics was dominated by two intertwined storylines. The first is that the acquisition of the FCSI mutation allowed SARS-CoV-2 to efficiently infect TMPRSS2-expressing airway epithelial cells at their cell surface and spread by respiratory transmission. The second is that the FCSI mutation crippled spike protein structure, trafficking, and function, and thereby established the conditions for subsequent emergence of an intragenic suppressor of FCSI’s deleterious traits, the D614G mutation. If this hypothesis is accurate, it is unlikely that WT SARS-CoV-2 would replicate for long in any environment, as passage in a TMPRSS2-dependent system would select for intragenic suppressor mutations such as D614G, while passage in a TMPRSS2-independent system would select for FCSI reversion mutations such as ΔN679-A688 and ΔR683-S689 (*24*). As for the hotly debated question of how the FCSI mutation arose (*42*, *84*–*87*), our data are only relevant to the relative timing of the FCSI and D614G mutations, and that they likely occurred in quick succession.

In addition to shedding new light on the evolutionary-genetic relationships between the FCSI and D614G mutations, our findings highlight the need to broaden the genetic and functional context in which spike mutations are considered. Specifically, while all spike mutations may represent adaptations to their environment, their analysis should also consider the alternative, which is that they arose to suppress deleterious effects of prior spike mutations. Moreover, while there is no doubt that spike mutations of interest should be tested for effects on spike-receptor interactions or spike-catalyzed membrane fusion, our results demonstrate that they should also be tested for their effects on spike protein trafficking and lysosome reprogramming.

### Implications for spike structure and trafficking

The observation that D614G enhances the trafficking of spike to lysosomes, reduces the cell surface expression of spike, and enhances spike-induced lysosome reprogramming adds to the already extensive array of D614G-associated phenotypes, which include increased loading of spike into virus particles, reduced loss of S1, increased thermal stability of spike, increased propensity to attain an up or “open” conformation, altered (or not altered) affinity for ACE2, and enhanced infectivity at early time points (*65*–*69*). As for how a single–amino acid substitution could affect such a diverse array of phenotypes, the most parsimonious explanation is that D614G restores an element of spike protein folding that was disrupted by the FCSI mutation and furin cleavage.

Existing structural models of WT and D614G spike trimers demonstrate that the D614G mutation affects the structure of uncleaved spike, which represents ~50% of spike proteins present on infectious virions (*5*, *68*). However, these models do not reveal the defining structural consequences of the FCSI mutation and furin cleavage that drove the pandemic, much less how the D614G mutation alters the structure of furin-cleaved spike, because all current structures are of spike proteins that lack a functional cleavage site. As a result, our existing structural models tell us little about the structural consequences of furin cleavage that triggered the SARS-CoV-2 pandemic, or how these changes are suppressed/reversed by the D614G mutation that so greatly accelerated this pandemic. We do not mean to trivialize the many challenges in determining spike protein structures, but the simple truth is that we will never know the actual effects of the FCSI and D614G mutations until structures can be obtained of spike proteins free of artificial mutations.

**Fig. 10.**
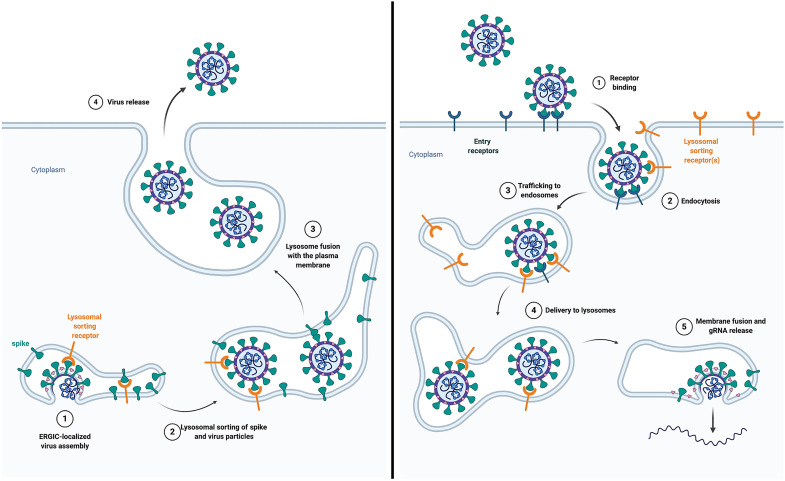
Endolyosomal model of SARS-CoV-2 egress and entry. We hypothesize that an as-yet-unidentified lysosomal sorting receptor for spike provides a common mechanistic link between the trafficking of spike to lysosomes during the biogenesis and release of SARS-CoV-2 virus particles (left) and the rapid postreceptor delivery of virus particles to endolysosomal compartments (right). Furthermore, we hypothesize that the FCSI mutation disrupted the structure of spike’s ECD, thereby inhibiting both egress and entry, while D614G at least partly reversed these defects.

While the structural consequences of the FCSI and D614G mutations remain largely unknown, there is no doubt that the FCSI mutation disrupted the trafficking of spike to lysosomes and also the TMPRSS2-independent pathway of virus infectivity. Given that intragenic suppressors are nearly always allele and mechanism specific (*88*), the suppression of both defects by the D614G mutation suggests that they are linked by a common mechanism, as does the fact that FCSI suppressor and reversion mutants both redirect spike from the plasma membrane to lysosomes. As for the mechanisms that link these processes, we propose that it lies in the endolysosomal model of SARS-CoV-2 egress and entry (Fig. 10). The basic features of this model were evident from previous studies (*25*–*38*, *89*), and we add to them only by hypothesizing the existence of a lysosome sorting receptor for spike that (i) mediates the lysosomal accumulation of newly synthesized spike proteins and (ii) facilitates a post-receptor, endolysosomal delivery of spike-decorated virus particles. Under this model, the FCSI mutation and furin cleavage inhibit both of these processes, while D614G restores both. Our proposal of a lysosomal sorting receptor for spike is highly speculative but it is not without precedent, as the endolysosomal egress and entry of varicella zoster virus is mediated by interactions of viral surface proteins and mannose-6-phosphate receptors (*90*). However, we do not know anything about how spike is trafficking to lysosomes other than the fact that it occurs, is mediated by its ECD, and is not mediated by the AP-2 and AP-3 pathways that deliver most membrane proteins to lysosomes. It should also be noted that the sorting differences between WT spike and D614G spike are a matter of degree, as both proteins can be found at both lysosomes and the plasma membrane, both proteins are able to drive lysosome reprogramming, and both proteins would therefore presumably be able to support the lysosomal egress of SARS-CoV-2.

### Spike drives several hallmarks of virus-induced lysosome reprogramming

Another important finding of this study is that it suggests a role for spike in the infected cell and, more specifically, in reprogramming lysosomes and other endomembrane systems in ways that match what occurs in virus infected cells. Prior studies have identified orf3a as the primary mediator of lysosome deacidification, lysosome exocytosis, and secretion of lysosomal hydrolases (*18*, *19*). Our observation extends those earlier reports by showing that spike expression alone is sufficient to drive lysosome clustering, the lysosomal accumulation of KDEL receptors, and the aberrant secretion of KDEL-containing ER resident proteins. These observations expand the scope of spike’s functions to include the remodeling of lysosome structure and function in infected, virus-producing cells. Although we do not yet know how spike’s reprogramming of lysosome structure and function affects viral biology, the association of these roles with viral infection and their augmentation by the fitness-enhancing D614G mutation indicates that these questions warrant further investigation.

### Limitations of this study

The major limitation of this study is that we did not identify spike’s lysosomal sorting receptor or show how spike protein structure is altered by the FCSI mutation and restored by the FCSI suppressor mutation D614G. Furthermore, we did not identify the specific subregion of spike’s ECD that mediates its trafficking to lysosomes nor did we interrogate the effect of the D614G mutation on host immune surveillance or immune responses, which might be affected by its pronounced effect on cell surface spike expression.

## MATERIALS AND METHODS

### Experimental design

The objective of this study was to test whether the D614G mutation affected the intracellular trafficking of SARS-CoV-2 spike and, if it did, whether its effects were (i) a specific adaptation to the new human host of SARS-CoV-2 or (ii) a specific adaptation to the only other major change in the virus, the FCSI mutation that occurred at nearly the same time as the zoonosis. The study was designed to study spike in isolation, apart from all other SARS-CoV-2 proteins, by expressing it and its mutant forms from an inducible promoter in a well-characterized human cell line [human embryonic kidney (HEK) 293] and then confirming our key observation in cells infected with G614 versus D614 strains of SARS-CoV-2.

### Cell lines, culture, transfection, and transgenic cell lines

Human HEK293 (CRL-1573) and opossum OK (CRL-1840) cell lines were obtained from the American Type Culture Collection. 293F cells were obtained from Thermo Fisher Scientific (R79007). VeroE6/TMPRSS2 cells were a gift from the National Institute of Infectious Diseases, Japan. HTet1 cells, a “Tet-on” derivative of HEK293 cells, FTet1 cells, a “Tet-on” derivative of 293F cells, Htet-derived cell lines, Ftet1-derived cell lines, and OK-derived cell lines were maintained in complete medium [Dulbecco’s modified Eagle’s medium high glucose, with glutamine, containing 10% fetal bovine serum (FBS) and 1% penicillin/streptomycin solution], supplemented with transgene-selecting antibiotics as needed, at 37°C, 90% H_2_O, and 5% CO_2_. For measurement of spike-induced changes in the secretion of ER-resident proteins, Ftet1, FTet1/S^W1^, and FTet1/S^D614G^ cell lines were cultured in shaker flasks in CDM (Freestyle) at 110 rpm, 37°C, 90% H_2_O, and 8% CO_2_. Spike protein expression was in all cases induced by addition of doxycycline to the culture medium at a final concentration of 1 μg/mL. Transfections were performed using Lipofectamine 2000 or 3000 according to the manufacturer’s instructions.

Transgenic cell lines were created as follows. To make the “Tet-on” HTet1 and FTet1 cell lines, HEK293 cells and 293F cells were transfected with the plasmid pS147. Two days after transfection, the cells were placed in zeocin-containing medium (final concentration of 200 μg/ml) and fed every 3 to 5 days, followed by pooling of all zeocin-resistant cell lines to generate the polyclonal cell lines HTet1 and FTet1. Derivatives of these cell lines were generated by transfecting HTet1 or FTet1 cells with pITRSB-based Sleeping Beauty transposons (*92*) carrying (i) a puromycin resistance gene and (ii) a spike-expressing gene. Two days after transfection, the cells were split into medium containing puromycin (2 μg/mL). Medium was changed every 3 to 5 days for 10 to 14 days, and after all nonresistant cells had died, the cells were pooled in medium containing both zeocin and puromycin and expanded to create the HTet1/S^W1^, HTet1/S^D614G^, HTet1/S^W1^-CSM cell lines, HTet1/S^W1^-ΔN679-A688, and HTet1/S^W1^-ΔR683-S689 cell lines, as well as the FTet1/S^W1^, FTet1/S^D614G^, and FTet1/S^W1^-CSM cell lines. OKTet1/S^W1^ and OKTet1/S^D614G^ cells were created by transfecting OK cells with pITRSB transposons that delivers three genes: (i) a constitutively expressed puromycin resistance gene, (ii) a doxycycline-regulated spike-expressing gene, and (iii) a doxycycline-induced rtTAv16-expressing gene.

### Viruses

VeroE6/TMPRSS2 cells (*41*) were used to grow and titrate infectious virus using established protocols in a P3 facility (*91*, *92*). The clinical isolates SARS-CoV-2/USA/MD-HP00076/2020 (spike D614; GenBank: MT509475.1) and SARS-Cov-2/USA/DC-HP00007/2020 (spike G614; GenBank: MT509464.1) were isolated using published procedures (*75*) from samples obtained through the Johns Hopkins Hospital network. For virus working stocks, VeroE6/TMPRSS2 cells grown in a T75 or T150 flask were infected at an MOI of 0.001 with virus diluted in medium. After 1-hour incubation at 33°C, the inoculum was removed, and medium was added (10 ml for T75 flask and 20 ml for a T150 flask). When cytopathic effect was seen in approximately 75% of the cells, the supernatant was harvested, clarified by centrifugation at 400*g* for 10 min, aliquoted, and stored at −65°C. Virus stocks were sequenced to confirm that the amino acid sequence of the isolate was identical to the sequence derived from the clinical sample. Virus-infected cells were analyzed by flow cytometry and IFM within the P3 facility or were fixed within the P3 facility and then interrogated under normal laboratory conditions.

### Plasmids

The plasmid pS147 carries a single gene consisting of the cytomegalovirus (CMV) enhancer/promoter sequences upstream of a single open reading frame encoding (i) rtTAv16 (*93*), (ii) the porcine teschovirus 2a peptide, and (iii) a codon-optimized bleomycin-resistance gene (*94*), followed by the expression-enhancing woodchuck hepatitis virus post-transcriptional regulatory element (WPRE) (*95*) and a polyadenylation signal. Sleeping Beauty transposons were based on pITRSB (*94*). pCG145, pCG200, and pCG298 are paralogous pITRSB vectors that are designed to transfer a transposon carrying two genes: (i) a puromycin-resistance gene (*94*) under the control of the EF-1α‌ short (EFS) promoter and (ii) a spike-expressing gene under control of the doxycycline-inducible TRE3G promoter upstream, with the only difference being that pCG145 encodes WT spike, or S^W1^, identical to the spike reference sequence (National Center for Biotechnology Information Reference Sequence YP_009724390.1). pCG200 is nearly identical to pCG145 except that it carries the D614G mutation in its spike gene and expresses S^D614G^, pCG298 is nearly identical to CG145 except that it expresses S^W1^-CSM, and pCG595 and pCG596 are nearly identical to CD145 except that they express S^W1^-ΔN679-A688 and S^W1^-ΔR683-S689, respectively. In addition, we created derivatives of pCG145 and pCG200 in which a TRE3G-rtTAv16 gene was inserted at the 3′ end of the transposon, followed by use of the resulting vectors (pCG308 and pCG310) to generate the OKTet/S^W1^ and OKTet/S^D614G^ cell lines.

### Antibodies, probes, drugs, and human plasmas

Affinity-purified antibodies specific for the C-terminal 14 amino acids of spike (-DSEPVLKGVKLHYT_COOH_) were raised in both rabbits and in sheep, and their specificity for spike in HEK293 cell lysates was established by immunoblot (fig. S2). Other primary antibodies were obtained from SinoBiologicals (anti-spike S2 1A9), Thermo Fisher Scientific [specific for ERGIC3 (#16029-1-AP), ERGIC53 (#13364-1-AP), calnexin (#PA5-34665), EEA1 (#48453), and Lamp2 (MA1-205)], Cell Signaling Technology [mTOR (#2972S)], BD Biosciences [GM130 (#610822) and CD81 (#555675)], Novus [CD63 (#NBP2-32830)], BioLegend [CD9 (#312102)], Abcam [SARS-CoV-2 spike S2 1A9 (#ab273433), BiP/GRP78 (#21685), and Lamp1 (#24170)] or were gifts from N. Altan-Bonnet (monoclonal antibodies to BiP/GRP78 and Lamp1 and a polyclonal antibody to KDEL receptors). Fluorescent and horseradish peroxidase (HRP) conjugates of anti-mouse, anti-rabbit, anti-human, and anti-sheep immunoglobulin G were obtained from Jackson ImmunoResearch. A647-dextran [10,000 molecular weight (MW); #D22914] was obtained from Thermo Fisher Scientific. The furin protease inhibitor decanoyl-RVKR-CMK was obtained from Tocris (#3501). Selleckchem was the source for dynasore (#S8047), pitstop2 (#S2970), and nocodazole (#S2775), which were used at final concentrations of 50, 20, and 1 μM, respectively.

COVID-19 patient plasmas were collected using standard procedures for blood draw and plasma collection. Following Johns Hopkins Medicine Institutional Review Board (IRB) approval, plasma samples were obtained under informed consent from healthy donors before the COVID-19 pandemic (JHM IRB NA_0004638) (*96*) and from patients with COVID-19, with specimens used for this publication obtained from the Johns Hopkins Biospecimen Repository, which is based on the contribution of many patients, research teams, and clinicians, and were collected following IRB approval [Johns Hopkins COVID-19 Clinical Characterization Protocol for Severe Infectious Diseases (IRB00245545) and Johns Hopkins COVID-19 Remnant Specimen Repository (IRB00248332)]. All COVID-19 patient plasmas used in this study were collected on the day of admission of the patient into the Johns Hopkins Hospital and between the dates of 7 April and 22 April 2020.

### Immunoblot

Cells were grown in the presence or absence of doxycycline, lysed by addition of SDS–polyacrylamide gel electrophoresis (PAGE) sample buffer, boiled for 10 min, separated by SDS-PAGE, and transferred to polyvinylidene difluoride membranes. Membranes were incubated with gentle rocking in blocking solution [5% nonfat dry milk in tris-buffered saline (pH 7.4) containing 0.1% Tween 20 (TBST)] at room temperature for 2 hours (blocking solution containing primary antibodies overnight at 4°C), washed five times in TBST, incubated with HRP-conjugated secondary antibodies in blocking solution for 1 hour at room temperature, and then washed five times with TBST. Membranes were then incubated with Amersham ECL Western Blotting Detection Reagents to generate HRP-mediated chemiluminescence and visualized immediately using an Amersham Imager 600 gel imaging system.

### Biotin labeling and analysis

HTet1/S^W1^ and HTet1/S^D614G^ cells were grown overnight in the presence of doxycycline to induce expression of the WT and D614G forms of spike. Cells were then chilled to 4°C, incubated at this temperature in phosphate-buffered saline (PBS) containing sulfo-NHS-biotin (Pierce) for 30 min, to covalently attach biotin to exposed primary amines of proteins at the surface of the HTet1/S^W1^ and HTet1/S^D614G^ cells. The protein labeling reaction was terminated by excess primary amine, the cells were washed, and lysates of surface-labeled HTet1/S^W1^ and HTet1/S^D614G^ cells were incubated with avidin-coupled beads for 30 min. The flow through was discarded, the beads were washed, and the biotin-labeled, avidin-bound proteins were eluted in SDS-PAGE sample buffer. Equal amounts of biotin-labeled cell surface proteins from HTet1/S^W1^ and Htet1/S^D614G^ cells were processed for immunoblot using antibodies specific for spike and control proteins.

### Flow cytometry

For flow cytometry of 293 cells expressing spike, we used the following protocol. Before the day of experiment, 100 μg of anti–SARS-CoV-2/S2 (1A9) antibody was conjugated to A647 using the Lightning-Link Conjugation Kit (Abcam, catalog no. ab269823) according to the manufacturer’s protocol. A total of 0.5 million cells were seeded on a six-well plate, grown in complete medium containing doxycycline (final concentration of 1 μg/mL) for 20 hours, dissociated with TrypLE Express (Thermo Fisher Scientific), passed through a cell-strainer cap (Falcon, catalog no. 352235), transferred to an Eppendorf tube, and spun at 500*g* for 4 min at 4°C. The resulting cell pellet was resuspended in 100 μL of chilled fluorescence-activated cell sorting (FACS) buffer (1% FBS in PBS) containing 2 μl of the A647 conjugated 1A9 antibody and incubated on ice in dark for 30 min with gentle flicking every 10 min. Cells were then washed three times by adding 1 mLof chilled FACS buffer each time, spinning at 500*g* for 4 min at 4°C, and discarding the supernatant. After the final wash, cells were resuspended in 250 ml of chilled FACS buffer containing DAPI (0.5 mg/ml), incubated on ice in dark for 5 min, and analyzed using a CytoFLEX S flow cytometer (Beckman Coulter). Cells were gated on the basis of (i) forward scatter area (FSC-A) versus side scatter area (SSC-A) (P1), (ii) FSC-A versus forward scatter height (FSC-H) (P2), (iii) FSC-A versus Pacific Blue 450 area (PB450-A) (P3), and (iv) FSC-A versus allophycocyanine area (APC-A) (P4). Approximately 20,000 singlet, live cells (after gate P3) were recorded on the APC channel (A647 fluorescence), and the positive signals (after gate P4) were analyzed by the percentage of parent (P4/P3) and mean APC-A at P4.

For flow cytometry of virus-infected cells, we used the following protocol. A total of 1 × 10^5^ cells were seeded on a six-well plate and grown in complete medium for 24 hours. Cells were then infected with HP07 (D614G) or HP76 (WT) at an MOI of 1. At 18 hours after infection, the cells were then washed and dissociated with TrypLE Express (Thermo Fisher Scientific), neutralized using stop solution (10% FBS in PBS), transferred to an Eppendorf tube, and spun at 500*g* for 5 min at 4°C. The resulting cell pellet was resuspended in 1000 μl of FACS buffer (1% FBS in PBS) containing LIVE/DEAD Aqua cell stain (Thermo Fisher Scientific) for 30 min in the dark. For the surface-stained cells, the samples were washed, blocked, and incubated with A647-conjugated 1A9 antibody and incubated on ice in dark for 30 min. Cells were then washed and fixed for 20 min. For intracellular staining, cells were washed, fixed, and permeabilized for 20 min. The samples were washed and then blocked (7% bovine serum albumin in PBS) for 30 min. Cells were then incubated with A647-conjugated 1A9 antibody for 30 min. Cells were then washed three times by adding 500 μl of FACS buffer each time, spinning at 500*g* for 4 min at 4°C, and discarding the supernatant. After the final wash, cells were resuspended in 700 μl of FACS buffer and filtered through a 35-μm strainer cap into FACS tubes. Cell suspensions were run on a BD LSRII Flow Cytometer using DIVA software. Single-stained cells were used as controls, and fluorescence minus one control was used to assist in gating. Data analysis was completed on FlowJo V10. Cells were gated on the basis of (i) FSC-A versus SSC-A (P1), (ii) FSC-A versus FCS-H (P2), and (iii) FSC-A versus Live Dead Stain AQUA (P3). Approximately 50,000 singlet, live cells (after gate P3) were recorded.

### Immunofluorescence microscopy

293 cell lines were cultured on sterile, poly-l-lysine–coated cover glasses in complete medium or in complete medium containing drugs, vehicle, or probes. At the appropriate time point for each experiment, cells were fixed (4% formaldehyde in PBS) for 15 min, permeabilized (1% Triton X-100 in PBS) for 5 min, and processed for IFM by incubation with primary antibodies for 20 min, followed by five washes in PBS, and incubation with secondary antibodies for 20 min, followed by five washes in PBS. For IFM of vacuolin-1–treated cells, cover glass–grown HTet1/S^D614G^ cells were switched from complete medium to complete medium containing doxycycline, grown overnight to allow expression of spike, followed by addition of vacuolin-1 at a concentration of 10 mg/ml for 1 hour, then fixed, and processed as above. For A647-dextran incubation, cover glass–grown HTet1/S^D614G^ cells were incubated in complete medium containing doxycycline and A647dextran (final concentration of 200 mg/ml), grown for 10 hours, washed, incubated in medium lacking A647-dextran for 3 hours, then fixed, and processed as above. For virus experiments, VeroE6/TMPRSS2 cells were grown on chamber slides, infected at an MOI of 10, incubated for 18 hours, then fixed and processed as described above.

Cover glasses of fixed and processed cells were mounted on slides using Fluoromount-G (Electron Microscopy Sciences). Cells were visualized using an EVOSM7000 fluorescence microscope equipped with 20× (PL FL 20×, 0.50 NA/2.5 WD), 40× (PLAN S-APO 40×, NA 0.95, 0.18 mm), and 60× (OBJ PL APO 60×, 1.42 NA/0.15 WD) Olympus objectives. Confocal fluorescence microscopy was performed using a Zeiss LSM800 microscope with gallium-arsenide phosphide (GaAsP) detectors and a 100×/1.4 NA Plan-Apochromat objective. Images were assembled into figures using Adobe Photoshop and Adobe Illustrator. Images were analyzed using ImageJ, in combination with the Robust Automatic Threshold Selection (RATS) plugin. With RATS, we segmented both the Lamp2 and spike channels. A binary mask was generated from each channel, and then colocalized regions were calculated as region of interest (ROI). The average gray value of ROI was measured.

### Electron microscopy

HTet1/SD614G cells were grown in doxycycline-containing complete medium on coated tissue culture plates for 2 days. The cells were then fixed with formaldehyde and glutaraldehyde, dehydrated, embedded in Epon, sectioned, interrogated with rabbit anti-spike C-terminal peptide antibody and monoclonal anti-Lamp2 antibody, and washed. Sections were then incubated with 12-nm gold conjugates of goat anti-rabbit antibodies—also with 12-nm gold conjugates of goat anti-rabbit antibodies and 6-nm gold conjugates of goat anti-mouse antibodies—washed, and stained with uranyl acetate. Sections were imaged on a Hitachi 7600 transmission electron microscope.

### Protein secretion assays

FTet1, FTet1/S^W1^, and FTet1/S^D614G^ cells were seeded at a density of 1 million cells/ml in 150-ml shaker flasks and grown at a shaking speed of 110 rpm for a period of 3 days in 30 mL of FreeStyle 293 Expression Medium (Thermo Fisher Scientific) supplemented with 1% penicillin/streptomycin solution and doxycycline (1 μg/mL). Cell pellets were collected by centrifugation at 300*g* for 5 min and lysed in 2 mL of 2× SDS-PAGE sample buffer (with 2-mercaptoethanol, Halt protease inhibitor cocktail, and phosphatase inhibitor cocktails 2 and 3). For the conditioned medium, large cell debris was removed by centrifugation at 3000*g* for 15 min, and the supernatant was passed through a 0.22-μm filter and spun at 100,000*g* for 2 hours to remove extracellular vesicles. The resulting supernatant was concentrated to 100 μL using a Centricon Plus-70 centrifugal flow concentrator (10-kDa cutoff; Millipore, catalog no. UFC701008) and lysed in 50 mL of 6× SDS-PAGE sample buffer (with 2-mercaptoethanol, Halt protease inhibitor cocktail, and phosphatase inhibitor cocktails 2 and 3) to generate the extracellular protein lysates for immunoblot analysis.

### Statistical analysis

Quantitative results were evaluated using Student’s *t* test (two-tailed, unequal variances) using GraphPad Prism version for Windows.

## Notes

View/request a protocol for this paper from *Bio-protocol*.
