# Supplementary material for: The D614G mutation redirects SARS-CoV-2 spike to lysosomes and suppresses deleterious traits of the furin cleavage site insertion mutation
Source: Sci Adv. 2022 Dec 23;8(51):eade5085. doi: 10.1126/sciadv.ade5085 (PMC9788772; doi:10.1126/sciadv.ade5085)
Supplement: Supplementary file 1 — Figs. S1 to S5 [file sciadv.ade5085_sm.pdf]

Science Advances
AAAS

Supplementary Materials for

The D614G mutation redirects SARS-CoV-2 spike to lysosomes and suppresses deleterious traits of the furin cleavage site insertion mutation

Chenxu Guo *et al.*

Corresponding author: Stephen J. Gould, sgould@jhmi.edu

Sci. Adv. **8**, eade5085 (2022)
DOI: 10.1126/sciadv.ade5085

This PDF file includes:

Figs. S1 to S5

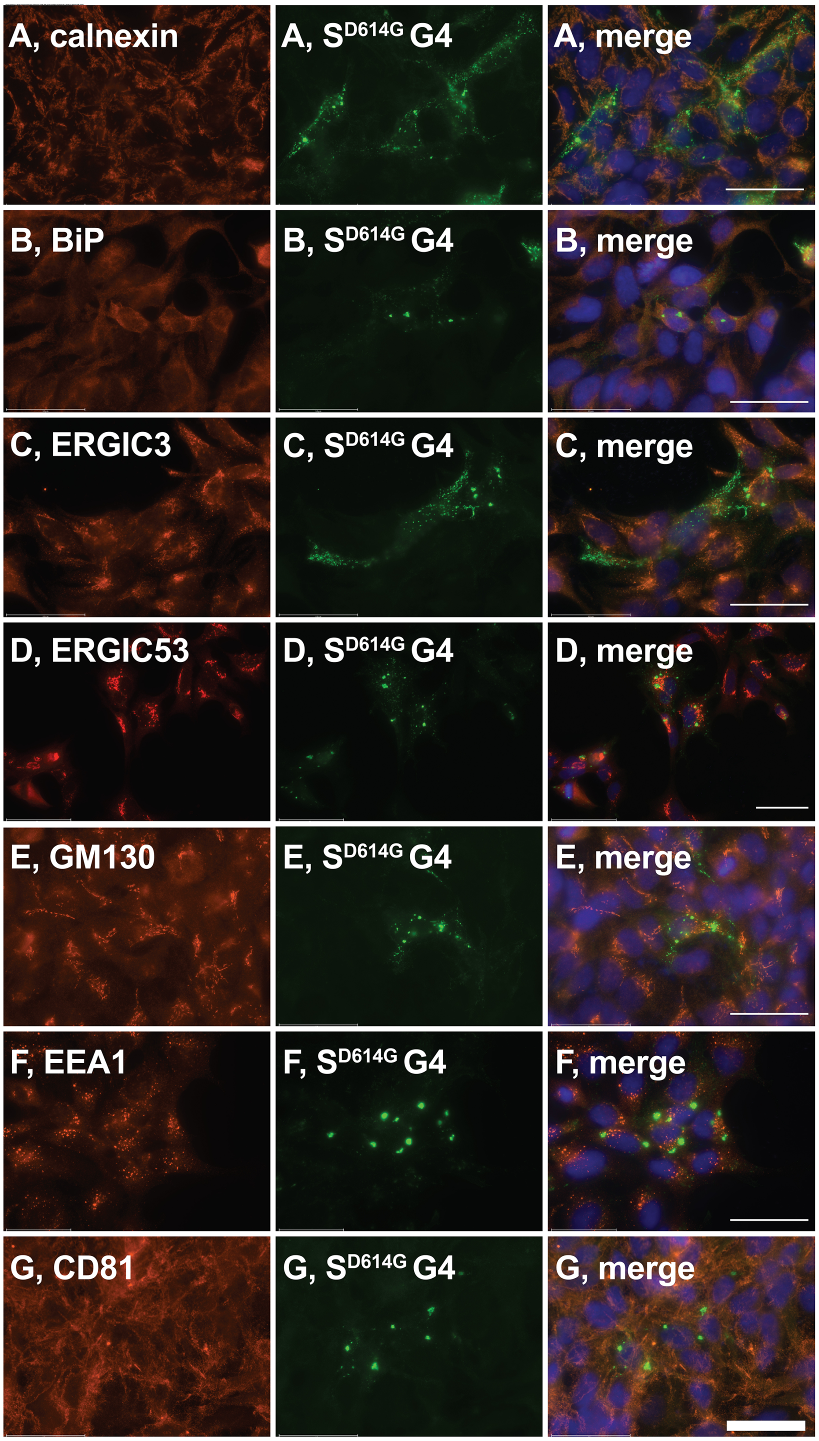

Fig. S1. SARSCoV-2 Spike does not co-localize with markers of most organelles. Fluorescence micrographs of doxycycline-induced Htet1/S^{D614G} cells that had been fixed, permeabilized and stained using (green) anti-Spike immune sera, (blue) DAPI, and (red) antibodies specific for (A) calnexin, (B) BiP/GRP78, (C) ERGIC3, (D) ERGIC53, (E) GM130, (F) EEA1, or (G) CD81. Bar, 50 mm.

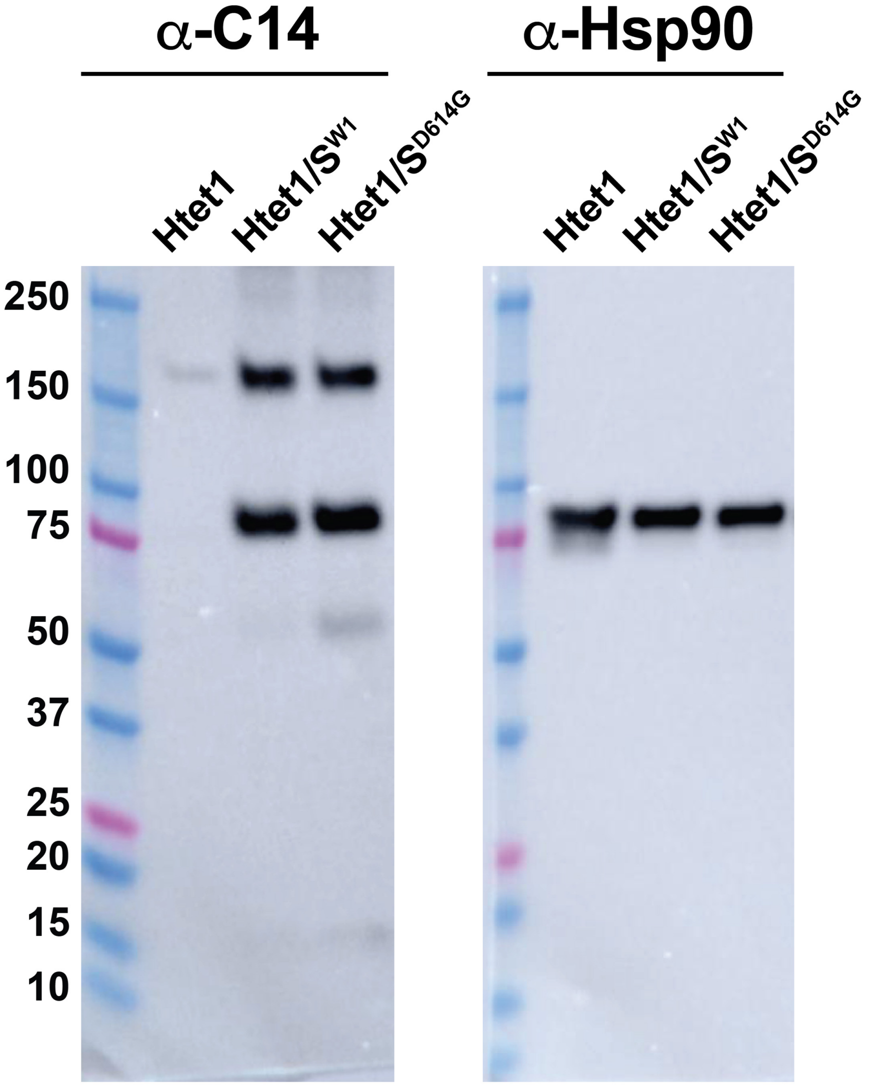

Fig. S2. Specificity of the anti-Spike C14 antibody. Immunoblot analysis of cell lysates generated from doxycycline-induced Htet1, Htet1/S^{W1} and Htet1/S^{D614G} cells probed with (left panel) affinity-purified antibodies raised against the C-terminal 14 amino acids of SARS-CoV-2 Spike and (right panel) anti-Hsp90 antibodies. MW size markers, from top, in kDa: 250, 150, 100, 75 (pink), 50, 37, 25 (pink), 20, 15, 10.

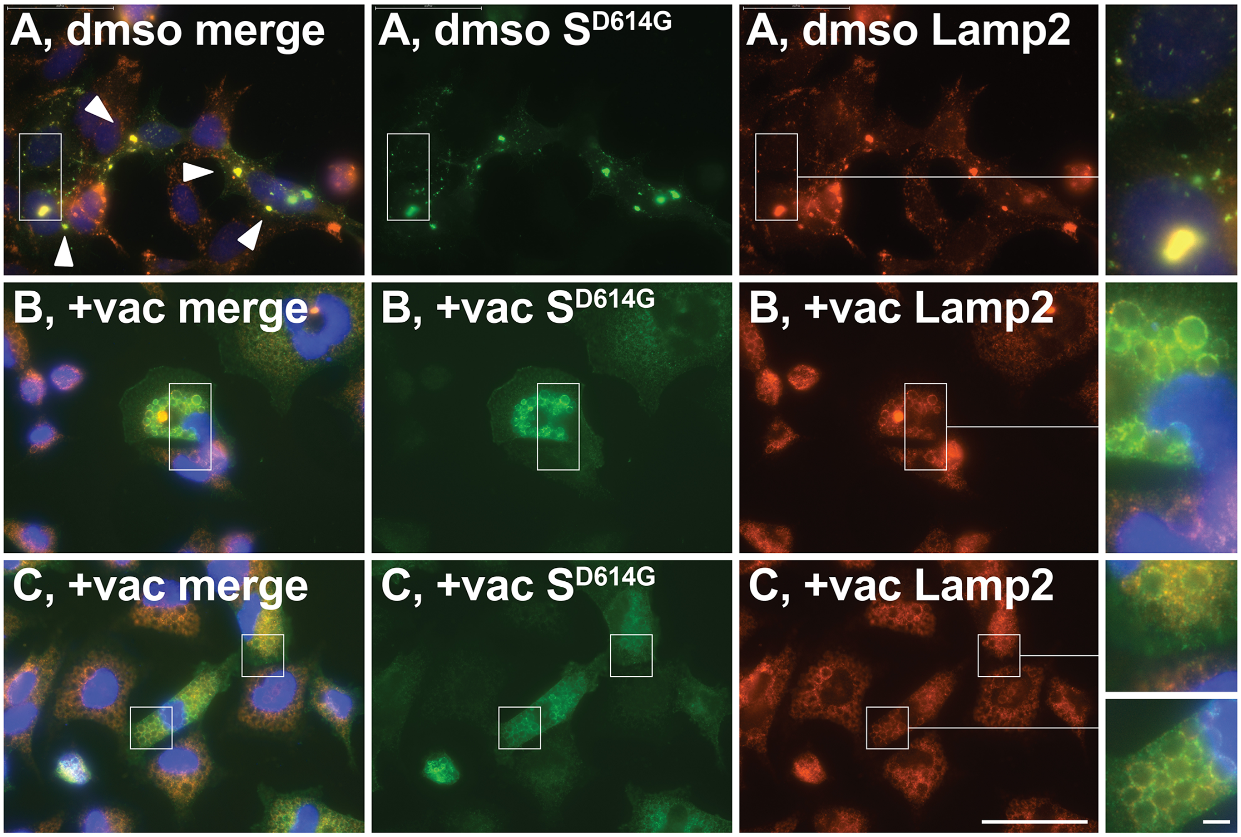

Fig. S3. Spike co-localizes with Lamp2 in vacuolin-treated cells. Fluorescence micrographs of doxycycline-induced Htet1/S^{D614G} cells that had been incubated for 3 hours with either (A) DMSO or (B, C) vacuolin-1, then fixed and processed for immunofluorescence microscopy using (green) anti-Spike immune sera, (blue) DAPI, and (red) an anti-Lamp2 antibody. Bar in main images, 50 um. Inset (3.2-fold higher magnification) shows greater detail in an area of particular interest. Bar in inset, 4 um.

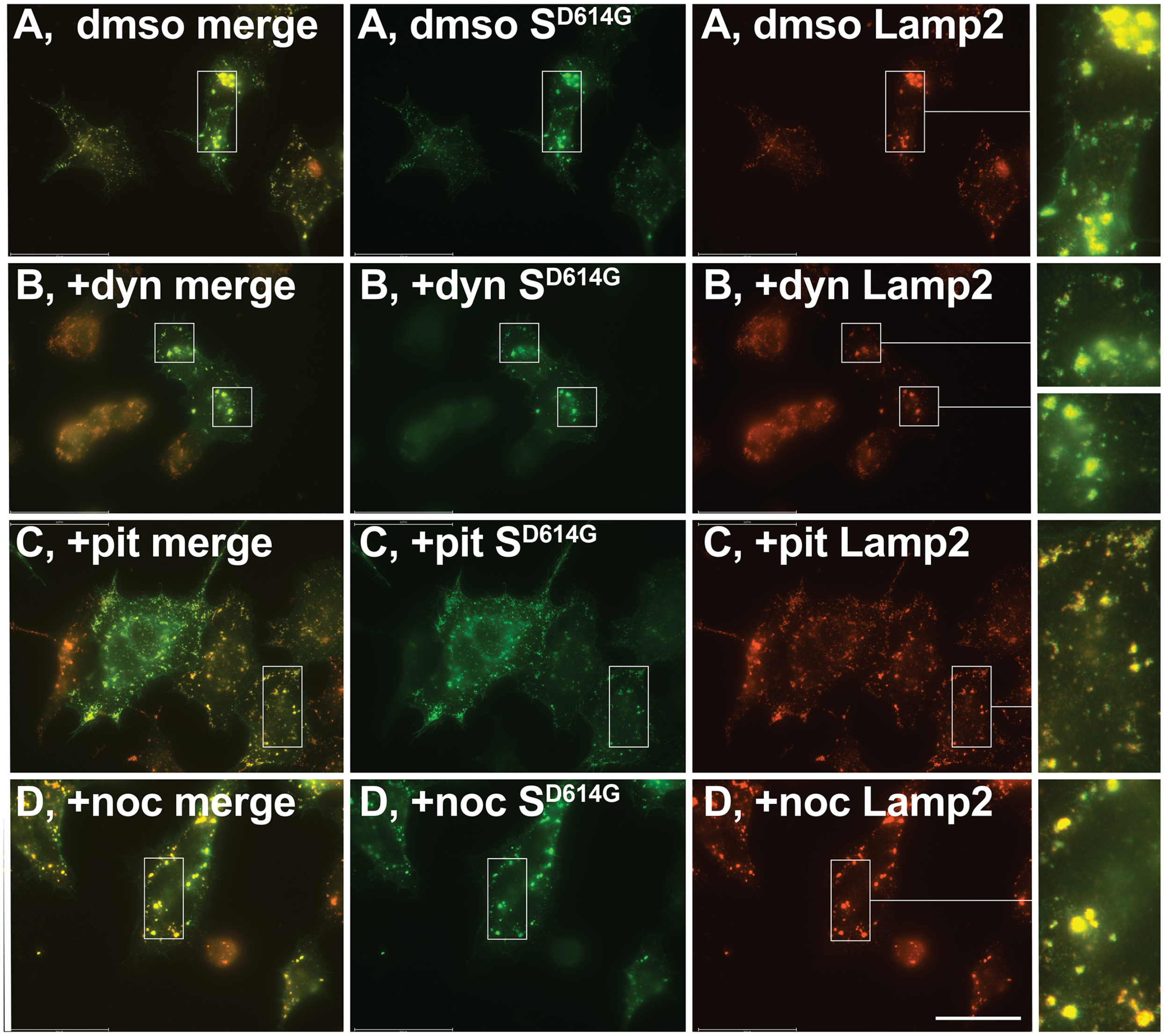

Fig. S4. Dynasore, pitsopt2 and nocodazole do not prevent Spike trafficking to lysosomes. Fluorescence micrographs of Htet1/S^{D614G} cells that had been incubated with doxycycline and (**A**) DMSO, (**B**) dynasore, (**C**) pistop2, and (**D**) nocodazole overnight, then fixed, permeabilized and stained using (green) COVID-19 serum G4, and (red) anti-Lamp2 antibodies. Bar, 50 um. Outsets (3.2-fold higher magnification) show greater detail in areas of particular interest.

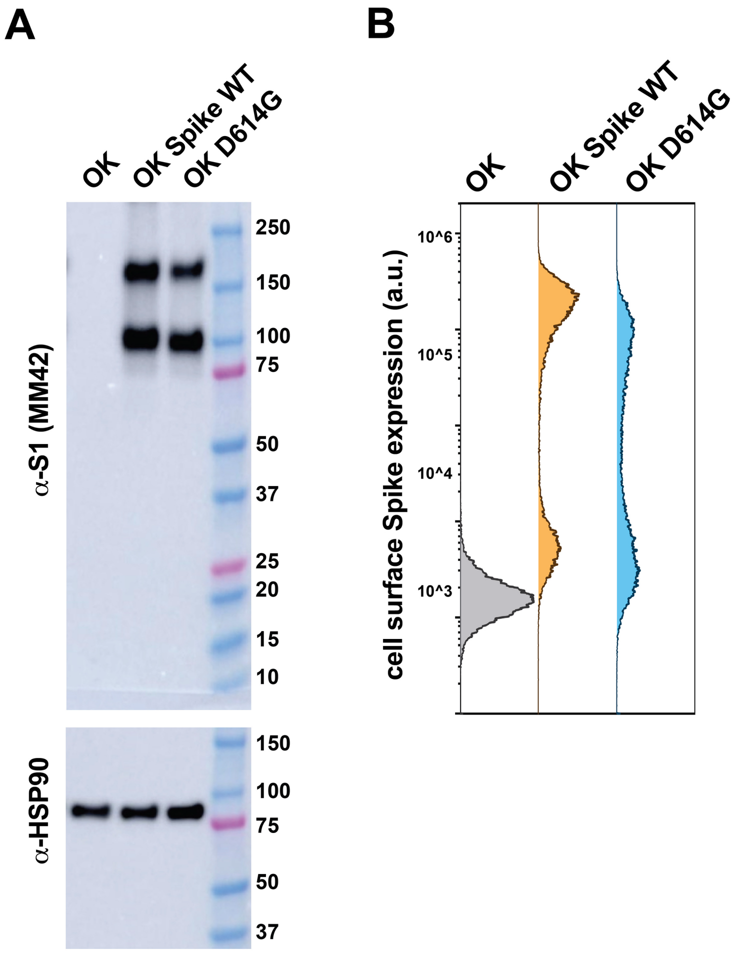

C

cell line	surface Spike (a.u.)
OK	0
OKtet/S^{W1}	1
OKtet/S^{D614G}	0.3

Fig. S5. The D614G mediated shift on Spike protein trafficking is not a human adaptation. (**A**) Anti-Spike and anti-HSP90 immunoblots of cell lysates prepared from equal amounts of doxycycline-induced OK cells, OK/S^{W1} cells, and OK/S^{D614G} cells. MW markers in kDa, from top, are 250, 150, 100, 75 (pink), 50, 38, 25 (pink), 20, 15, and 10. (**B**) Histograms of cell surface anti-Spike fluorescence measurements captured by flow cytometry of doxycycline-induced (grey) OK cells, (orange) OK/S^{W1} cells, and (blue) OK/S^{D614G}. (**C**) Relative cell surface expression of Spike in each of the cell lines tested in these experiments. Similar results were observed in 3 independent rials (n = 3).
